# Supplementary material for: α-Synuclein-induced deformation of small unilamellar vesicles
Source: QRB Discov. 2022 Jul 25;3:e10. doi: 10.1017/qrd.2022.9 (PMC10392696; doi:10.1017/qrd.2022.9)
Supplement: Supplementary file 1 [file S2633289222000096sup001.pdf]

Supporting Information: α-synuclein-induced deformation of small unilamellar vesicles

Katarzyna Makasewicz, Stefan Wennmalm, Sara Linse, Emma Sparr

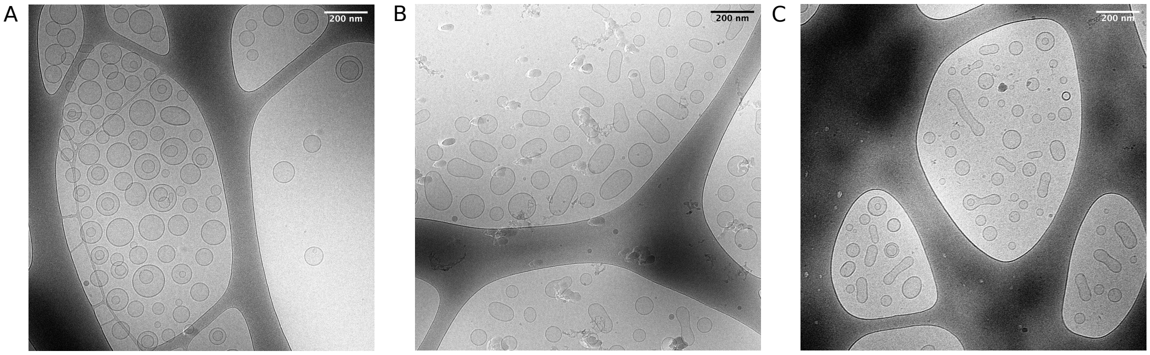

Figure S1: Cryo-TEM images of A) free DOPC:DOPS 7:3 small unilamellar vesicles (SUVs) B) DOPC:DOPS 7:3 SUVs with α-synuclein at L/P 200 25 s after the addition of the protein. C) DOPC:DOPS 7:3 SUVs with α-synuclein at L/P 200 24 h after the addition of the protein.

Sample	Particles per frame	Standard error
free SUVs	67,6	3,3
SUVs + α-synuclein at L/P 25	69	4,9
SUVs + α-synuclein at L/P 50	62	1,4
SUVs + α-synuclein at L/P 100	65	2.3
SUVs + α-synuclein at L/P 200	67.1	3.1

Table S1: Nanoparticle Tracking Analysis of DOPC:DOPS 7:3 SUVs with and without α-synuclein at different L/P ratios. All the samples contained 500 nM lipids and varying concentrations of α-synuclein. Each sample was measured 3 times, with each measurement consisting of 5 runs of 30 s. The measured region of the sample was changed for every run.

Inverse Fluorescence Cross-Correlation Spectroscopy

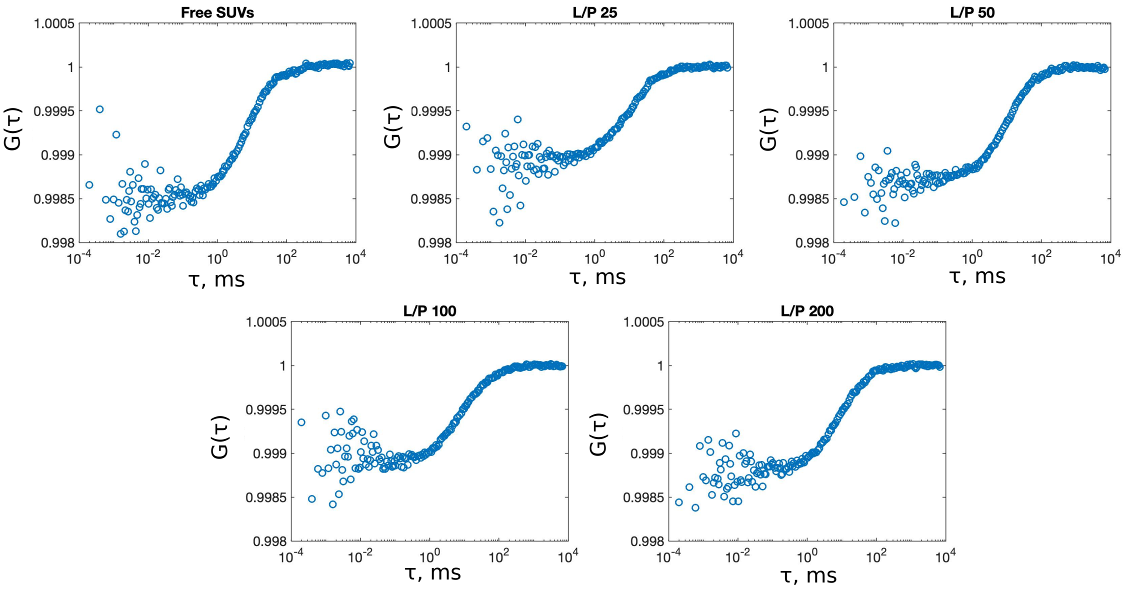

Figure S2: Anti-correlation curves from the inverse fluorescence cross-correlation experiment for free SUVs and SUVs with α-synuclein at L/P 25, 50, 100 and 200.

The theory and fundamental principles of Inverse FCS (iFCS) and Inverse FCCS (iFCCS) have been described previously [1, 2]. Since in iFCCS, a high fluorescence signal of several MHz is recorded from the fluorophores surrounding the particles of interest and cross-talk mainly occurs from the green to the red channel, it can be advantageous to use red dyes for the surrounding solution and green labeled particles of interest. Though this reduces the amount of cross-talk, it still exists and needs to be corrected for.

Below, the relationship between the particle volume and the amplitude of the iFCCS function is derived.

The amplitude of the cross-correlation function is given by:

$$G_{cc}(0) - 1 = \frac{dI_g(0)dI_r(0)}{I_g I_r} \quad (1)$$

where I_g and I_r are the mean detected fluorescence intensities in the green and the red channels, and dI_g and dI_r are the deviations from those mean values. The detected fluorescence intensity in the red channel is given by:

$$I_r = I_{r,tot}(1 - N_{p,r}V_q) + N_{p,r}\frac{V_g}{V_r}q_{p,g}CT \tag{2}$$

where $I_{r,tot}$ is the red fluorescence intensity when there are no particles in the detection volume, $N_{p,r}$ is the number of particles in the red detection volume, $V_q = V_p/V_r$ where V_p is the volume of a particle, V_r is the red detection volume, V_g is the green detection volume, $q_{p,g}$ is the brightness of the particles in the green channel, and CT (cross-talk) is the fraction of the total detected green signal that is detected in the red channel. Derivation with respect to $N_{p,r}$ gives:

$$\frac{dI_r}{dN_{p,r}} = -I_{r,tot}V_q + \frac{V_g}{V_r}q_{p,g}CT \tag{3}$$

Since the fluctuations of the particle number is a Poissonian process it follows that $\Delta N_{p,r}$ = $(N_{p,r})^{\frac{1}{2}}$ and:

$$dI_r = (\frac{V_g}{V_r}q_{p,g}CT - I_{r,tot}V_q)N_{p,r}^{\frac{1}{2}} \tag{4}$$

The detected fluorescence intensity in the green channel, I_g, is given by:

$$I_g = N_{p,g}q_{p,g} = N_{p,r}\frac{V_g}{V_r}q_{p,g} \tag{5}$$

where $N_{p,g}$ is the number of particles in the green detection volume, which equals $N_{p,r}V_g/V_r$ from scaling with the ratio of the two detection volumes. Derivation with respect to $N_{p,r}$ gives:

$$\frac{dI_g}{dN_{p,r}} = \frac{V_g}{V_r}q_{p,g} \tag{6}$$

and with $\Delta N_{p,r} = (N_{p,r}^{\frac{1}{2}})$ it follows that:

$$dI_g = \frac{V_g}{V_r} q_{p,g} N_{p,r}^{\frac{1}{2}} \tag{7}$$

Inserting equations (2), (4), (5), and (7) into equation (1) gives:

$$G_{cc}(0) - 1 = \frac{\frac{V_g}{V_r} q_{p,g} N_{p,r}^{\frac{1}{2}} (\frac{V_g}{V_r} q_{p,g} CT - I_{r,tot} V_q) N_{p,r}^{\frac{1}{2}}}{N_{p,r} \frac{V_g}{V_r} q_{p,g} (I_{r,tot}(1 - N_{p,r} V_q) + N_{p,r} \frac{V_g}{V_r} q_{p,g} CT)} \tag{8}$$

which after after simplification becomes:

$$G_{cc}(0) - 1 = \frac{\frac{V_g}{V_r} q_{p,g} CT - I_{r,tot} V_q}{I_{r,tot}(1 - N_{p,r} V_q) + N_{p,r} \frac{V_g}{V_r} q_{p,g} CT} \tag{9}$$

Since 1-$N_{p,r} V_q \approx 1$ and $I_{r,tot} >> N_{p,r} \frac{V_g}{V_r} q_{p,g} CT$, equation (9) can be approximated by:

$$G_{cc}(0) - 1 = \frac{\frac{V_g}{V_r} q_{p,g} CT}{I_{r,tot}} - V_q \tag{10}$$

In our measurements, $\frac{V_g}{V_r} = 0.6$, $q_{p,g}$ =50 kHz, CT =0.01, and $I_{r,tot} \approx 3000$ kHz. Thus:

$$V_q = \frac{V_p}{V_r} = \frac{\frac{V_g}{V_r} q_{p,g} CT}{I_{r,tot}} - (G_{cc}(0)) - 1) = \frac{0.6 \cdot 50 \cdot 0.01}{3000} - (G_{cc}(0) - 1) = 10^{-4} - (G_{cc}(0) - 1) \tag{11}$$

As an example, the amplitude of the curve in figure 4B is -0.0017 (0.9983-1) and -0.00221 after correction for detector dead time (see section below). Together with V_r=$0.3 \cdot 10^{-15}$ L, this gives that V_p=$7.0 \cdot 10^{-19}$ L. For spherical particles this corresponds to a mean radius of 55 nm.

iFCS dead time correction

As has been described previously [3], the fact that the photon detectors are not linear at count rates of several MHz affects the amplitude of the auto- and cross-correlation curves, and thereby the estimated particle volumes. This is because the signal when there are no particles in the focus is higher, and therefore the detector is more saturated, compared to when a particle is in the focus, and this affects the observed depth of the negative intensity spikes. The GaAsP detectors used in the Zeiss 780 and 980 LSMs have a dead time of 66 ns, and the corrected count rate is described by:

$$CR_0 = \frac{CR}{1 - t_d CR} \tag{12}$$

where CR_0 is the corrected count rate, CR is the measured count rate, and t_d is the detector dead time. In our measurements we kept the total count rate in the red channel as close to 3 MHz as possible. At this count rate the corrected amplitude of the cross-correlation curve in iFCCS (and thereby the particle volume) is 30 % larger than that observed.

References

[1] Stefan Wennmalm, Per Thyberg, Lei Xu, and Jerker Widengren. Inverse-fluorescence correlation spectroscopy. *Analytical chemistry*, 81(22):9209–9215, 2009.

[2] Stefan Wennmalm and Jerker Widengren. Inverse-fluorescence cross-correlation spectroscopy. *Analytical chemistry*, 82(13):5646–5651, 2010.

[3] Tor Sandén, Romain Wyss, Christian Santschi, Ghérici Hassaïne, Cédric Deluz, Olivier JF Martin, Stefan Wennmalm, and Horst Vogel. A zeptoliter volume meter for analysis of single protein molecules. *Nano letters*, 12(1):370–375, 2012.
